# The Alberta population-based prospective evaluation of the quality of life outcomes and economic impact of bariatric surgery (APPLES) study: background, design and rationale

**DOI:** 10.1186/1472-6963-10-284

**Published:** 2010-10-08

**Authors:** Raj S Padwal, Sumit R Majumdar, Scott Klarenbach, Dan W Birch, Shahzeer Karmali, Linda McCargar, Konrad Fassbender, Arya M Sharma

**Affiliations:** 1 Department of Medicine, University of Alberta, Edmonton, Alberta, Canada; 2 Department of Surgery and CAMIS (Center for the Advancement of Minimally Invasive Surgery), University of Alberta, Royal Alexandra Hospital, Edmonton, Alberta, Canada; 3 Department of Agricultural, Food and Nutritional Sciences, University of Alberta, Edmonton, Alberta, Canada; 4 Department of Oncology, University of Alberta, Edmonton, Alberta, Canada

## Abstract

**Background:**

Extreme obesity affects nearly 8% of Canadians, and is debilitating, costly and ultimately lethal. Bariatric surgery is currently the most effective treatment available; is associated with reductions in morbidity/mortality, improvements in quality of life; and appears cost-effective. However, current demand for surgery in Canada outstrips capacity by at least 1000-fold, causing exponential increases in already protracted, multi-year wait-times. The objectives and hypotheses of this study were as follows: 1. To serially assess the clinical, economic and humanistic outcomes in patients wait-listed for bariatric care over a 2-year period. We hypothesize deterioration in these outcomes over time; 2. To determine the clinical effectiveness and changes in quality of life associated with modern bariatric procedures compared with medically treated and wait-listed controls over 2 years. We hypothesize that surgery will markedly reduce weight, decrease the need for unplanned medical care, and increase quality of life; 3. To conduct a 3-year (1 year retrospective and 2 year prospective) economic assessment of bariatric surgery compared to medical and wait-listed controls from the societal, public payor, and health-care payor perspectives. We hypothesize that lower indirect, out of pocket and productivity costs will offset increased direct health-care costs resulting in lower total costs for bariatric surgery.

**Methods/design:**

Population-based prospective cohort study of 500 consecutive, consenting adults, including 150 surgically treated patients, 200 medically treated patients and 150 wait-listed patients. Subjects will be enrolled from the Edmonton Weight Wise Regional Obesity Program (Edmonton, Alberta, Canada), with prospective bi-annual follow-up for 2 years. Mixed methods data collection, linking primary data to provincial administrative databases will be employed. Major outcomes include generic, obesity-specific and preference-based quality of life assessment, patient satisfaction, patient utilities, anthropometric indices, cardiovascular risk factors, health care utilization and direct and indirect costs.

**Discussion:**

The results will identify the spectrum of potential risks associated with protracted wait times for bariatric care and will quantify the economic, humanistic and clinical impact of surgery from the Canadian perspective. Such information is urgently needed by health-service providers and policy makers to better allocate use of finite resources. Furthermore, our findings should be widely-applicable to other publically-funded jurisdictions providing similar care to the extremely obese.

**Trial Registration:**

Clinicaltrials.gov NCT00850356

## Background

Obesity currently affects 24% of Canadians[1] and is a chronic medical condition that leads to substantial morbidity,[2] premature mortality,[3] impaired quality of life (QOL),[4] and increased health care costs [5]. Obesity is most commonly defined according to body mass index (BMI), with BMI levels of 30-34.9, 35-39.9 and over 40 kg/m^2 ^corresponding to Class I, II and III obesity, respectively. Extreme obesity, defined herein as patients with moderate or severe obesity, is the fastest growing obesity subgroup affecting nearly 8% of Canadians [6]. Extreme obesity has increased in prevalence in Canada by 400% in two decades;[5] increases the risk of type 2 diabetes by up to 18-fold compared to normal-weight individuals;[2] shortens life expectancy by 8-13 years;[7] increases work-absenteeism;[8] and dramatically reduces QOL,[4] productivity,[9] and employability [10]. Health care expenditures in the 3% of the employed US population that are severely obese account for 21% of all health care costs associated with obesity [11].

### Bariatric Surgery for Extreme Obesity

Lifestyle modification (diet, exercise ± behavioural therapy) and pharmacotherapy for obesity each reduce weight by approximately 3-5% but are limited by poor long-term effectiveness and sub-optimal adherence [12,13]. In comparison, bariatric surgery leads to substantial weight reduction and has emerged an effective means to reduce weight and improve comorbidity in patients with extreme obesity [14]. Surgery is currently indicated in medically refractory patients with BMI levels of ≥ 40 kg/m^2 ^or BMI levels of ≥ 35 kg/m^2 ^with a major obesity-related comorbidity (e.g., hypertension, diabetes, sleep apnea) [14].

Bariatric procedures either involve stomach restriction alone or combined restriction plus intestinal diversion. The types of procedures performed have evolved over the past several decades and certain procedures, such as banded gastroplasty, have been abandoned due to poor long-term weight loss results. In Canada and globally, the most common operations performed are adjustable gastric banding (42%), Roux-en-y gastric bypass (40%) and sleeve gastrectomy (5%). Ninety percent of bariatric procedures are performed via laparoscopic (minimally invasive) techniques [15]. In gastric banding, the proximal stomach is encircled with an adjustable band that is progressively inflated to create a small, restrictive gastric pouch which reduces meal portions. In the roux-en-y gastric bypass, a highly restrictive gastric pouch is created and coupled with diversion of the upper small intestine. The sleeve gastrectomy procedure is performed by fashioning the stomach into an elongated tube and resecting the majority of the greater curve of the stomach.

### Outcomes Associated with Bariatric Surgery

Although no large scale, contemporary randomized controlled trials (RCTs) have examined the impact of surgery on cardiovascular morbidity or overall mortality, compelling data are available from high quality observational studies such as the Swedish Obesity Study (SOS), a matched cohort study of 2010 surgical and 2037 controls [16,17]. Compared to the poor long-term results of non-surgical therapy, surgery is the only therapy associated with substantial improvements in weight (averaging 33% after 2-3 years[18] and 16% after 10 years[16]); 15-year mortality rates (5.0% versus 6.3% in well-matched controls; HR 0.71; 95% CI 0.54 to 0.92);[19] 11-year incidence rates of first time cancers (HR 0.67; 95% CI 0.53-0.85);[17] and 7-year mortality rates from coronary artery disease (HR 0.41; 95% CI 0.21-0.78) and cancer (HR 0.40; 95% CI 25-0.65) [20]. In terms of other medical comorbidity, surgery increased remission rates for type 2 diabetes (73% versus 13%; p < 0.001; OR 5.5; 95% CI 2.2-14) in a 60-patient RCT;[21] and meta-analyses of primarily observational data has demonstrated that surgery is associated with resolution or improvement of type 2 diabetes, hypertension, dyslipidemia and sleep apnea in 70-86% of cases [18]. Additional studies demonstrate that surgery significantly (p < 0.05) improves psychosocial functioning,[22] quality of life,[23] and physical function [24].

In terms of Canadian data, a retrospective analysis of 1035 bariatric surgery patients from a single practice in Quebec reported 5-year excess weight losses of 61-75% following gastric bypass and banding [25]. In an earlier study, patients from this bariatric program (n = 1035) were also retrospectively compared with 5746 age and sex-matched controls identified using administrative data claims in Quebec (clinical variables such as height and weight were not available for controls) [26]. Mortality rates over 5 years were markedly lower in the bariatric surgery cohort compared to controls (0.68% versus 6.17%; RR 0.11, 95% CI 0.04-0.27) although the design of this study cannot rule out the very real likelihood that surgical selection bias (i.e., healthier and higher socioeconomic status patients with lower likelihood of surgical complications more likely to receive surgery) explains some if not most of these findings.

### Risks of Surgery

The complications of surgery can be divided into peri-operative and long-term complications. The totality of data suggests that the benefits of surgery far outweigh these risks [18,27]. Perioperative death rates are 0.1-0.5% and immediate postoperative complications (e.g., clots, cardiorespiratory events and wound infections) occur in 10% of individuals. Diversionary procedures increase the long-term risk of nutrient deficiency (up to 50% of patients) while gastric bands can slip (6% of patients) or erode (10%) necessitating re-operation [27]. Some long-term consequences of surgery such as micronutrient deficiencies (e.g., vitamin D deficiency and metabolic bone disease) are incompletely understood and require further study.

### Economic Evaluations of Bariatric Surgery

From the payor perspective and relative to the commonly cited thresholds of acceptability,[28,29] the long-term (20 years to lifetime) cost-effectiveness of surgery compared with non-surgical management appears attractive. Incremental cost-effectiveness ratios (ICERs) range from $5000 to $35 000 per quality-adjusted-life-year (QALY) [30]. The cost-effectiveness of surgery in patients with type 2 diabetes appears dominant (provides net health benefits and cost savings) compared to non-surgical interventions [31,32]. A recent Canadian economic evaluation performed by our group from the health care payor's perspective estimated that surgery is associated with ICERs of $8000-10 000/QALY over a lifetime horizon in Canada, with more favourable ICERs in subjects with greater obesity related comorbidity [30]. The only other additional published economic data from the Canadian perspective have been reported from a retrospective cohort study from Quebec. Surgery reduced health care utilization for a variety of disorders and was cost saving after 3.5 years compared to matched controls identified through administrative data claims [33].

However, there are limitations to the above studies. Economic studies based solely upon administrative data inputs do not include home and workforce productivity and patient borne costs. Furthermore, cost effectiveness analyses from the payor perspective do not examine such costs. Therefore, these additional data elements would provide a much more accurate picture of overall costs and benefits from a societal perspective.

### Demand, Access and Wait Times For Bariatric Surgery

Demand for bariatric surgery has increased at an exponential rate. The number of procedures performed globally has increased from 5000 in 1987-9 to 350 000 (63% in US/Canada) in 2009 [15,34]. In Canada, the annual number of procedures performed in public health care facilities in the past decade has increased nearly 19-fold to ~1500 procedures per year [35,36].

Despite this dramatic rise in uptake, the number of individuals potentially eligible for surgery greatly exceeds current surgical capacity. Given that the number of Canadians potentially eligible for surgery is 5.8% or 1.5 million (assuming a 2009 adult population of nearly 26 million),[37] and that about 1500 procedures are performed annually in Canada,[36] only 0.1% of potentially eligible patients are accessing surgery in this country. Therefore, actual demand may be orders of magnitude greater than current provision of surgery [38].

In Canada and elsewhere, surgery is available in both publicly and privately funded programs. Private surgery costs approximately $17 000 in Canada and is unaffordable to many [39]. However, wait times for publicly funded bariatric procedures in this country average 5 years,[40] and are similarly protracted in other public health care systems [41]. This clearly indicates a substantial demand-supply gap. A 2005 Ontario Ministry of Health report estimated demand at 3500 surgeries per year in that province, a 7-fold higher number than the 500 surgeries currently performed annually [42]. This necessitated outsourcing of procedures to the US at substantial cost;[42] and resulting in petitions from advocacy groups demanding improved access [43]. In 2009, Ontario announced $75 million funding to increase procedure numbers from 244/year to nearly 1500/year [38].

### Knowledge Gaps

In summary, the prevalence of extreme obesity has increased dramatically and bariatric surgery is the most effective treatment available. However, access to bariatric care in Canada is severely limited and wait times are lengthy. The ramifications of protracted wait times on health and health care costs have not previously been examined. In addition, data assessing the clinical and cost effectiveness of surgery in Canada are limited and further study is needed. Specifically, a comprehensive, population-based prospective assessment of the economic consequences from a health care payor, public payor (health care + other benefits such as unemployment insurance and other transfer payments), and societal (public payor, out-of-pocket costs, home and work productivity costs) perspective has not been performed to our knowledge in Canada or elsewhere.

Lastly, much of the prospective data evaluating bariatric procedures comes from the SOS study. However, vertical banded gastroplasty, which is now outdated, comprised nearly 70% of the procedures performed in SOS. More recent studies have evaluated gastric banding and gastric bypass and have reported similar results to SOS in terms of weight reduction and improvement in obesity-related comorbidities [44,45]. However, no prior studies comparing sleeve gastrectomy to medical management and APPLES results will help address this knowledge gap.

## Objectives and Hypotheses

The APPLES study is a population-based, prospective controlled study enrolling at minimum of 500 patients and designed to assess the impact of extended wait-times for bariatric care and examine the clinical and cost-effectiveness of bariatric treatment in the Canadian context.

The three major objectives of APPLES are:

1. To assess the impact of extended wait times for bariatric care by examining the two-year change in clinical, economic and humanistic outcomes in wait-listed patients. We hypothesize that patients will report progressive deterioration in these outcomes over time.

2. To determine the clinical effectiveness and changes in health-related quality of life associated with modern bariatric procedures and compare these outcomes to medically treated controls and untreated wait-listed controls. We hypothesize that surgery will markedly reduce body weight, decrease the need for unplanned medical care, and increase quality of life compared to controls.

3. To compare the costs associated with bariatric surgery to costs associated with medical and wait-listed controls over three years, from societal, public payor, and health care payor perspectives. We hypothesize that lower indirect, out of pocket and productivity costs will offset increased direct health-care costs resulting in lower total costs for bariatric surgery.

## Methods/design

### Overall Study Design

**Figure 1:**
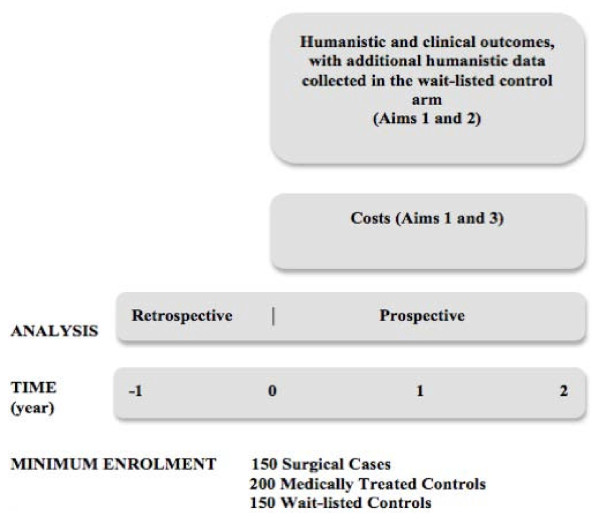
**APPLES Study Design**.

In this prospective cohort study, consecutive and consenting patients enrolled in the Weight Wise Regional Obesity Program and without a contraindication to surgery will be enrolled. The minimum enrolment sample size will include 150 surgical patients, 200 patients receiving intensive medical therapy and 150 patients wait-listed to enter the clinic (Figure 1). The number of patients enrolled in the medical arm is larger because we anticipate an increased rate of censoring in this study arm, as some of these patients will ultimately undergo bariatric surgery within the two-year follow-up period.

### Study Setting and Subject Recruitment

The Edmonton Weight Wise program is a comprehensive initiative established in 2005 designed to deliver integrated, patient-focused, evidence-based care to the Edmonton Zone of Alberta Health Services (AHS). This region is one of the largest integrated health delivery systems in Canada and includes a catchment population of approximately 1 million residents within greater Edmonton and an additional 600 000 residents in surrounding regions cared for by more than 1000 physicians, with an annual healthcare budget of almost two billion dollars [46].

**Figure 2:**
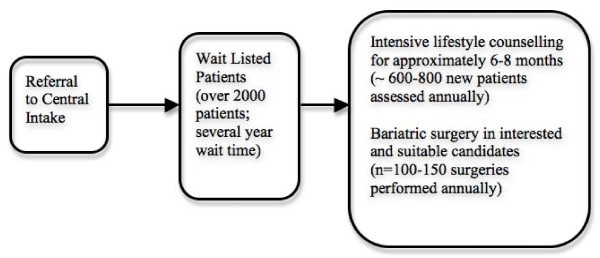
**Structure of the Adult Component of Weight Wise**.

Weight Wise includes a central, region-wide single-point-of-access referral system; community education and weight management sessions; and adult and pediatric bariatric specialty clinics. Adult specialty services are offered to patients with BMI levels of ≥ 35 kg/m^2 ^referred from a medical practitioner. By extrapolating from contemporary Canadian obesity surveillance data (i.e. ~8% of Canadians are moderately [BMI 35.0-39.9 kg/m^2^] or severely [> 40 kg/m^2^] obese), we estimate that over 125 000 adult patients within our region's catchment area has a BMI ≥ 35 kg/m^2 ^[6]. Community-dwelling patients referred for evaluation in the adult clinic are wait-listed at the time of referral (Figure 2). Currently, over 2000 adult patients are wait-listed for entry into the specialty clinic and their average wait may be up to several years in duration. Wait-listed patients are expected to attend community-based group education sessions prior to clinic entry. Otherwise, they receive no specific intervention.

### Eligibility For Surgery

Within the adult specialty clinic, patients receive approximately 24-36 weeks of intensive lifestyle counselling (diet, exercise, behavioural modification), delivered by a multidisciplinary staff (internists, dieticians, nurses, physiotherapists, and psychologists) according to current recommendations [14]. Patients are seen approximately every 4-8 weeks. Patients interested in bariatric surgery are also evaluated for this procedure by the same multidisciplinary staff. Patients deemed to be appropriate candidates are subsequently evaluated by a bariatric surgeon. Patients with BMI levels ≥ 35-39.9 kg/m^2 ^and a major medical comorbidity (e.g., hypertension, type 2 diabetes, sleep apnea) or BMI levels ≥ 40 kg/m^2 ^are considered potential candidates for surgery. Absolute contraindications to surgery include pregnancy, uncontrolled psychiatric disease, active substance abuse or smoking (patients are required to quit prior to surgery), an active eating disorder (anorexia or bulimia), and high-risk for surgery medical status (e.g. severe coronary artery disease). Because of limited data documenting the benefits of surgery in patients younger than 18 years of age and evidence for possible harm in patients over 60,[47] procedures are not performed in these age groups. In order to access surgery, patients are also required to demonstrate commitment to attend scheduled appointments and adhere to lifestyle modification and behavioural therapy.

### Inclusion Criteria

1. Male or female patients in the Weight Wise Regional Obesity Program

2. 18-60 years old

3. BMI levels ≥ 35 kg/m^2 ^and a major medical comorbidity or BMI levels ≥ 40 kg/m^2^

4. Able to provide informed consent

### Exclusion Criteria

1. Pregnant or nursing

2. Currently participating in an obesity-related clinical trial or in whom protein-sparing low calorie diet is planned.

3. Contraindication to bariatric surgery and/or weight loss

4. Unable or unwilling to complete questionnaires or expected to experience difficulty with attendance of visits or completion of study data

5. Any other medical, social or geographic condition which, in the opinion of the investigators, would not allow safe completion of the study protocol

### Surgical Procedures Performed

Roux-en-Y gastric bypass, gastric banding and sleeve gastrectomy are all performed. Surgical techniques have been previously detailed [30,48,49]. Gastric banding is performed using the Swedish Adjustable Gastric Band (SAGB) Realize I/II(tm) (Johnson & Johnson/Ethicon Endosurgery, Cincinnati, Ohio) and sleeve gastrectomies are created over a 50 Fr bougie using staple line reinforcement throughout. Initially, gastric bypass was performed by hand sewing the gastrojejunostomy over a 34 Fr orogastric tube and positioning of the roux limb was retrocolic. Over the past two years, the technique has been modified and a 21 or 25 mm circular stapler is now used for the gastrojejunostomy pouch, with antecolic positioning of the roux limb. The roux limb is ~100 cm in length. The entero-enterostomy is created using varying techniques, including a combination of linear staplers and sutured closure.

### Follow-up

Follow-up visits will be scheduled every 6 months for two years and will be performed in-person or by telephone if necessary. The final two year follow-up visit will be performed in-person.

### Data Collection and Outcome Measures (Additional File 1 and Table 1)

**Table 1:** Cost categories and details of source data for quantification and valuation of relevant costs

**Cost Category**^**1**^	Source Details	Units of Resources	Time Frame
1. Inpatient encounters	Administrative data	# of hospitalizations length-of-stay	Provided by fiscal year Referenced to time zero
2. Outpatient encounters	Administrative data	# encounters # procedures	Provided by fiscal year Referenced to time zero
3. Physician Fees	Administrative data	# encounters Provider specialty Service provided	Provided by fiscal year Referenced to time zero
4. Medications	Patient interview	Name Dosage, frequency & duration	Bi-annual
5. Weight Wise Clinic Visits	Administrative data and chart review	Personnel (nurses, dieticians, support staff) salaries Administrative and capital costs Supplies & equipment	Provided by fiscal year Referenced to time zero
6. Home Care & Long Term Care	Patient interview	Personnel (nurse, OT, PT, RT) salaries Disability aids (walker, bars, rails, etc.) Administrative and capital costs	Bi-annual
7. Transfer Payments	Patient interview	Unemployment insurance Disability benefits Assured Income for the Severely Handicapped (AISH)	Bi-annual
8. Employment status, absenteeism	Patient interview	Employment status in past year (# hours/week, # weeks) Absenteeism in past year (# days) Annual income (by quintile)	Bi-annual
9. Weight Loss Interventions	Patient interview	Weight loss program, meal replacements, physical trainer, exercise programs, alternative therapies, nutritional counselling, commercial program	Bi-annual
10. Mobility and Medical	Patient interview	Mobility aids, home modification/renovations, rehabilitation, paid personal assistance (household activities and home productivity, driving)	Bi-annual
11. Personal/Household Productivity	Patient interview as part of quality of life surveys	Capacity to perform household/domestic activities, personal care, and participate in leisure activities (Scalar) Paid/unpaid caregivers	Bi-annual

AHCIP = Alberta Health Care Insurance Plan. MACAR = Morbidity and Ambulatory Care Abstracting Record. DAD = Discharge Abstract Database. LOS = Length of stay. CIHI = Canadian Institute for Health Information. OT = occupational therapy. PT = physiotherapy. RT = respiratory therapy. SW = social worker.

^1^Health Care Payor perspective includes cost categories 1-6; Public Payor perspective includes cost categories 1-7 and Societal Payor perspective includes all costs.

After informed consent is obtained, baseline data collection for consenting patients will include the following: age, sex, race, marital status, employment status, household income quintile (≤ 20 K, 20-40 K, 40-60 K, 60-80 K, ≥80 K), general medical history and obesity-related comorbidities, smoking status (current, past, never), detailed current and past medications, weight, BMI, waist circumference, blood pressure, fasting lipids, fasting glucose, HbA1c, and liver enzymes.

Body weight will be measured using a validated, calibrated scale to the nearest 0.1 kilogram after the patient has emptied his/her bladder. Subjects will wear light indoor clothing with empty pockets and no shoes. Height will be measured using a wall-mounted stadiometre. A single reading taken using an automated blood pressure monitor and using an appropriately sized blood pressure cuff will be recorded with the subject seated in a chair and after five minutes of rest.

Repeat assessment of blood pressure, body weight, and cardiovascular risk factors (cholesterol profile, glycemic parameters) will be performed at 2 years.

Additional outcomes to be collected every 6 months will include:

1. **Household income and employment status**

2. **Quality of life and utility measurement**: Health related quality of life will be measured using validated, widely-used instruments. Generic and preference-based quality of life will be assessed using the Short Form-12 (SF-12[50]) and the EuroQol-5D (EQ-5D[51]) respectively. Obesity-specific quality of life will be assessed using the Impact of Weight on Quality of Life-Lite (IWQOL-Lite) [52].

3. **Patient satisfaction: **Satisfaction with medical care will be assessed using two questions, which will be rated on a 5-point Likert Scale:

a. The medical care I have been receiving is just about perfect

b. I am dissatisfied with some things about the medical care I received.

4. **Impact of Extended Wait Times: **The Waiting List Impact Questionnaire (WLIQ),[53] is a previously validated 47-item list of open-ended patient statements designed to assess the impact of extended wait-times in Canada for coronary bypass surgery and will be administered to wait-listed patients. This instrument has been modified for use in a bariatric population and items deemed not relevant to the bariatric setting were eliminated, resulting in a 40-item questionnaire. The instrument assesses general quality of life on a scale of 0-100 and also examines specific domains (physical stress, social support, frustration, employment status), which are serially evaluated by patients on a 5-point Likert scale. The degree of interest in bariatric surgery is specifically assessed.

5. **Edmonton Obesity Staging Score (EOSS)**:[54] EOSS is a recently proposed, preliminary staging system for obesity which is based upon the presence or absence of obesity-related comorbities. EOSS will be evaluated as a triage and prognostic tool within the APPLES cohort.

6. **Economic Data **(Table 1): Economic data will be collected for the year prior to enrolment and in the two years following enrolment. Two major data sources will be used to collect economic data.

a. **Administrative Data**: Linkage to AHS and Alberta Health and Wellness (AHW) administrative data sources will be performed according to previously described methods [55,56]. Because all permanent residents of Alberta are eligible for insurance by AHW and over 99.9% participate in this coverage, these administrative data will comprehensively capture patient-specific health care resource utilization. Data elements include vital statistics (mortality) and the following health care resources and costs: inpatient and outpatient encounters, physician billings, medical procedures, and emergency room visits (Table 1).

b. **Patient-Reported: **Second, in order to comprehensively capture all economic consequences and enable a societal perspective, each patient enrolled in the study will provide additional information retrospectively for the year prior to enrolment and prospectively for two years after enrolment on a bi-annual basis. Resource use will be identified through investigator and clinical experience as well as examination of comprehensive costs lists, and published literature (Table 1) [57]. There is no previously validated data collection instrument to measure these costs in obesity; thus, we have developed a comprehensive instrument using commonly used techniques [58,59]. Societal costs include costs of weight loss interventions (meal replacement, weight loss programs, alternative therapy, medications), mobility and medical costs (mobility aids, home modifications, rehabilitation, home care, housekeeping), workforce productivity (employment, absenteeism), home productivity (domestic chores, leisure activity, use of paid or unpaid caregivers) and transfer payments (unemployment insurance), and will be valued in accordance with Canadian guidelines [60].

### Statistical Analysis and Sample Size Considerations

#### Clinical, Humanistic and Economic Outcomes in Wait-listed Patients

This analysis specifically focuses on the wait-listed group and will examine two-year change scores in each relevant outcome using the appropriate statistical methodology. Analyses will be conducted in all wait-listed patients as well as those indicating a specific interest in surgery. Examples of clinical outcomes include anthropometric indices, blood pressure, lipid profile, glycemic control, diabetes prevalence. Examples of humanistic outcomes include QOL and patient satisfaction. Economic outcomes include total and categorical costs. For example, two-year change scores will be analysed for QOL domains (e.g. SF-12, IWQoL-Lite) using appropriately calibrated and constructed linear regression models and/or analysis of variance. Because these are self-reported data collected on a semi-annual basis, we will use a "last-value carried forward" approach to handle missing data as our primary analytic strategy.

##### Sample Size Considerations

The sample size of 150 subjects provides ample power to detect 2-year change scores and explore potential independent correlates of change. For example, for a 2-year change in SF-12 domains such as physical function, with baseline score of 31.6, SD 9,[61] a clinically important change in QOL of 5 points, two-sided alpha = 0.05, beta = 0.90, and a 30% attrition rate, 48 patients would be required. Similarly, to detect a 2-year change in IWQoL-Lite, with a baseline score of 28.7, SD 18.7,[61] a clinically important change in QOL of 10 points,[62] two-sided alpha = 0.05, beta = 0.90, and a 30% attrition rate, 51 patients would be required.

#### Comparing Clinical and Humanistic Outcomes in Patients Treated with Surgery to Medically Treated and Wait-listed Patients

Outcomes are similar to those listed above. Two-year mean changes in continuous variables will be compared between the surgical arm and medical or wait-listed controls using unpaired t-tests for continuous outcomes and chi-squared tests for dichotomous ones. Multivariable predictors of the 2-year change in a given outcome will be identified using appropriately constructed and calibrated covariate-adjusted linear regression models for continuous outcomes or logistic regression models for dichotomous ones. Patients who cross over to another study arm (e.g., medically treated patients who undergo surgery) will be censored at the point of cross over and a last-observation-carried-forward approach to missing data will be used for the primary analysis.

##### Sample Size Considerations

As an example, we calculated the sample size required to examine changes in body weight. Conservatively assuming a 2-year weight loss of 33% for surgical patients with a standard deviation of 17%,[18,27] a 25% 2-year difference in weight between surgical patients and intensively treated medical controls (an extremely conservative estimate, as this difference is generally several orders of magnitude),[18,27] a 2-sided α = 0.05, β = 0.9, and 30% attrition rate, we would require 13 patients.

#### Comparisons of Costs Over Three Years Between the Surgical Arm, Medically Treated Controls and Wait-listed Controls

First, descriptive statistics will be used to assess cumulative and incremental costs (by perspective and cost category) in each year using the arithmetic mean and 95% confidence interval for normally distributed variables and median with interquartile ranges for skewed variables. Within and between-group comparisons will be performed. Distributions of cost are often skewed and will be normalized, if necessary, by using log-transformations. Unadjusted analyses of costs will be performed using ANOVA; if cost cannot be normalized then nonparametric (e.g., Kruskal-Wallis) tests will be used. If log-transformation of costs is necessary, Duan's smearing estimator will be used to calculate appropriate error estimates after the data are re-transformed from the logarithmic scale [63].

Second, covariate controlled sequential multiple linear regression models will be constructed to determine the independent association of surgery (versus medical or wait-list controls) on each incremental cost outcome. Cumulative costs over time will be calculated using standardized methods,[64,65]and costs for average hypothetical patients will be calculated using the least squares means method, and adjusted for significant predictor variables for costs for medical and wait-listed controls.

Third, the net economic impact of increasing bariatric surgery rates from a societal, public payor, and health care payor perspective will be assessed using a mathematical model developed by our team. The costs determined above will be input, and the net economic impact of increasing rates of bariatric surgery from current rates (0.1% of eligible patients) to 0.5% (estimated current rates in US), 5% and 10% of all eligible patients will be calculated.

##### Sample Size Considerations

Using previous estimates,[66] we have adequate power to detect a difference of 20% or greater in costs between the surgical groups and the medical controls or the wait list controls, assuming an average 2-year total incremental cost of $12 183 in surgical patients, a standard deviation of $6000, two sided alpha = 0.05, and beta = 0.90.

### Extended Follow-up

Using anonymised and de-identified study identification numbers, we will link our study sample with provincial administrative databases so as to facilitate extended follow-up of health care utilization, clinical events, and costs. We have undertaken several previous studies linking clinical registry and cohort data with these high quality databases, and have previously reported greater than 96% linkage success with follow-up extending 5 years and beyond [55,67].

### Ethics Approval

APPLES has been approved by the Health Research Ethics Board of the University of Alberta.

## Discussion

In summary, APPLES is a prospective observational study that aims to address current knowledge gaps by examining the impact of wait times for bariatric care in a surgery eligible population and by generating prospective, population-based Canadian bariatric clinical, economic and humanistic outcome data. Protracted, multi-year wait times are a major concern within Canada's publicly funded health care system [40]. Therefore, APPLES will help clinicians and decision makers determine whether or not current wait times are contributing to a deterioration in health outcomes and/or increases in costs. If such findings are demonstrated, further analyses may help to identify predictors of worsening outcomes and this may potentially inform how best to triage patients and optimally allocate scarce bariatric surgery resources. The paucity of data on appropriate triage of patients for surgery has been identified as a major gap in current knowledge and is suggested as a priority for future studies [68].

An additional advantage of the APPLES study design is that, because subjects are enrolled from a regional program, the study will examine outcomes and costs on a population-wide basis rather than from a more selected source such as a single clinic or group of clinics. Thus, we feel that the results should both inform health care delivery in the Canadian context as well as be generalizable to other single-payor systems with universal health care coverage. Notably, lengthy wait times for surgery are not isolated to Canada and are present in publicly funded health care systems across the world [41].

APPLES will also provide a comprehensive assessment of the costs related to bariatric care and will examine contemporary procedures, including sleeve gastrectomy. Data will be collected from both administrative and patient sources within a health care delivery model that includes universal access and comprehensive capture of hospital and outpatient encounters. These high quality costing data will include an assessment of costs commonly viewed as 'indirect' in nature. Although the time frame for data collection is limited to 2 years, these data will also be useful to inform economic models and will thus enable assessment of cost-effectiveness over a longer time frame. This will be particularly useful in more accurately assessing the overall cost-effectiveness of bariatric surgery in Canada. For example, a finding of unequivocal cost savings or neutrality from a public payor perspective would indicate that bariatric surgery is "dominant" (cost saving with health benefits) over medical management, and expansion should be a priority.

As of May 2010, 95% of baseline enrolment within APPLES has been completed. Recruitment of all 500 patients is expected by June 2010, with two-year follow-up extending to June 2012. Final results for the APPLES analysis are anticipated by late 2012 or early 2013.

## Competing interests

DB has received honoraria for advisory boards, teaching and research from Ethicon Endo-Surgery Inc., a Johnson and Johnson company. All other authors had no competing interests to declare at the time of submission of this manuscript.

## Authors' contributions

RP drafted the initial study concept and all authors contributed to the study design. RP wrote the initial draft of the protocol and all authors provided input into revisions and approved the final draft.

## Pre-publication history

The pre-publication history for this paper can be accessed here:

http://www.biomedcentral.com/1472-6963/10/284/prepub

## Supplementary Material

Additional file 1 **APPLES Case Report Forms**. baseline and follow-up data collection forms for the APPLES study Click here for file
