# Supplementary material for: The Alberta population-based prospective evaluation of the quality of life outcomes and economic impact of bariatric surgery (APPLES) study: background, design and rationale
Source: BMC Health Serv Res. 2010 Oct 8;10:284. doi: 10.1186/1472-6963-10-284 (PMC2964692; doi:10.1186/1472-6963-10-284)
Supplement: Additional file 1 — APPLES Case Report Forms. baseline and follow-up data collection forms for the APPLES study [file 1472-6963-10-284-S1.PDF]

APPLES Study

Patient ID # 0 1 – ____ – __ **Patient Initials** ___ ___ ___

Site Number | Patient Number | Group | F M L

Date of Baseline Visit ____ / ____ / ________
dd mm yyyy

Consent

Date Informed Consent signed ____ / ____ / ________
dd mm yyyy

Consent administered by ________________
First Initial & Last Name

Demographics

Date of Birth ____/____/________
dd mm yyyy

Sex ☐ Male ☐ Female

Current Marital Status

☐ Married/Common-Law
☐ Widowed
☐ Separated/Divorced
☐ Not answered
☐ Single/Never Married

Current Highest Level of Education

☐ No high school
☐ Some post secondary
☐ Some high school
☐ Completed Post Secondary
☐ High school diploma
☐ Not answered

Current Employment Status *(check all that apply)*

☐ Employed full-time
☐ Employed casual/volunteer
☐ On Long-Term Disability
☐ Not answered
☐ Homemaker full-time
☐ Unemployed
☐ Retired
☐ Employed part-time
☐ On Short-Term Disability
☐ Other, specify ________________

Current Household Income *(before taxes & deductions)*

Although many health expenses are covered by health insurance, there is still a relationship between health and income. Please be assured that, like all other information you have provided, these answers will be kept strictly confidential.

What is your best estimate of the total income, before taxes and deductions, of all household members from all sources in the past 12 months?

☐ < 15,000
☐ 50,000 – 79,999
☐ 15,000 – 29,999
☐ ≥ 80,000
☐ 30,000 – 49,999
☐ Not answered

Ethnicity *(check all that apply)*

☐ Caucasian
☐ Hispanic
☐ Other, specify ____________
☐ African American
☐ Oriental
☐ Not answered
☐ Aboriginal
☐ South Asian *(e.g. East Indian)*

Page completed by ________ Date ____/____/______
Initials dd mm yyyy

©2008 EPICORE Centre

Fax completed forms to the EPICORE Centre (780) 492-6059 or 1-888-215-5474

APPLES Study

Patient ID #	0 1	–		–		Patient Initials			
	Site Number		Patient Number		Group		F	M	L

Medical History/Obesity-Related Comorbidities

Have you ever had, or do you currently have, any of the following **medical conditions**

		If Yes (check all that apply)	If Current
☐ Yes ☐ No	Impaired Glucose Tolerance	☐ Past ☐ Current	☐ Self Reported ☐ Diagnosed
☐ Yes ☐ No	Diabetes Mellitus	☐ Past ☐ Current	☐ Self Reported ☐ Diagnosed
☐ Yes ☐ No	Hypertension	☐ Past ☐ Current	☐ Self Reported ☐ Diagnosed
☐ Yes ☐ No	Dyslipidemia	☐ Past ☐ Current	☐ Self Reported ☐ Diagnosed
	Cardiovascular Disease		
☐ Yes ☐ No	Coronary	☐ Past ☐ Current	☐ Self Reported ☐ Diagnosed
☐ Yes ☐ No	Peripheral	☐ Past ☐ Current	☐ Self Reported ☐ Diagnosed
☐ Yes ☐ No	Cerebral	☐ Past ☐ Current	☐ Self Reported ☐ Diagnosed
☐ Yes ☐ No	Congestive Heart Failure	☐ Past ☐ Current	☐ Self Reported ☐ Diagnosed
☐ Yes ☐ No	Sleep Apnea	☐ Past ☐ Current	☐ Self Reported ☐ Diagnosed
	If Yes, on CPAP ☐ Yes ☐ No		
☐ Yes ☐ No	Hypoventilation	☐ Past ☐ Current	☐ Self Reported ☐ Diagnosed
	If Yes, on Oxygen ☐ Yes ☐ No		
☐ Yes ☐ No	Asthma	☐ Past ☐ Current	☐ Self Reported ☐ Diagnosed
☐ Yes ☐ No	Gastroesophageal Reflux Disease	☐ Past ☐ Current	☐ Self Reported ☐ Diagnosed
☐ Yes ☐ No	NAFLD *(Fatty Liver)*	☐ Past ☐ Current	☐ Self Reported ☐ Diagnosed
☐ Yes ☐ No	Gallbladder Disease or Gallstones	☐ Past ☐ Current	☐ Self Reported ☐ Diagnosed
☐ Yes ☐ No	Other Gastronintestinal		
	If Yes, specify ________________	☐ Past ☐ Current	☐ Self Reported ☐ Diagnosed
☐ Yes ☐ No	Venous Stasis or Leg Ulcers	☐ Past ☐ Current	☐ Self Reported ☐ Diagnosed
☐ Yes ☐ No	Osteoarthritis	☐ Past ☐ Current	☐ Self Reported ☐ Diagnosed
☐ Yes ☐ No	Back Pain	☐ Past ☐ Current	☐ Self Reported ☐ Diagnosed
☐ Yes ☐ No	Fibromyalgia	☐ Past ☐ Current	☐ Self Reported ☐ Diagnosed
☐ Yes ☐ No	Chronic Pain *(other than above)*		
	If Yes, specify ________________	☐ Past ☐ Current	☐ Self Reported ☐ Diagnosed
☐ Yes ☐ No	Chronic Renal Disorder	☐ Past ☐ Current	☐ Self Reported ☐ Diagnosed
☐ Yes ☐ No	Urine/Fecal Incontinence	☐ Past ☐ Current	☐ Self Reported ☐ Diagnosed
☐ Yes ☐ No	Polycystic Ovary Syndrome	☐ Past ☐ Current	☐ Self Reported ☐ Diagnosed
☐ Yes ☐ No	Hypothyroidism	☐ Past ☐ Current	☐ Self Reported ☐ Diagnosed
	Cancer History		
☐ Yes ☐ No	Gastrointestinal	☐ Past ☐ Current	☐ Self Reported ☐ Diagnosed
☐ Yes ☐ No	Reproductive	☐ Past ☐ Current	☐ Self Reported ☐ Diagnosed
☐ Yes ☐ No	Other		
	If Yes, specify ______________	☐ Past ☐ Current	☐ Self Reported ☐ Diagnosed
☐ Yes ☐ No	Lymphedema	☐ Past ☐ Current	☐ Self Reported ☐ Diagnosed

Page completed by ________ Date ____/____/_______
Initials dd mm yyyy

©2008 EPICORE Centre

Fax completed forms to the EPICORE Centre (780) 492-6059 or 1-888-215-5474

APPLES Study

Patient ID # 0 1 – ☐☐☐☐ – ☐☐ **Patient Initials** ☐☐☐

Site Number Patient Number Group F M L

Have you ever had, or do you currently have, any of the following **mental health conditions**

		If Yes (check all that apply)	If Current
☐ Yes ☐ No	Depression	☐ Past ☐ Current	☐ Self Reported ☐ Diagnosed
☐ Yes ☐ No	Bipolar	☐ Past ☐ Current	☐ Self Reported ☐ Diagnosed
☐ Yes ☐ No	Anxiety	☐ Past ☐ Current	☐ Self Reported ☐ Diagnosed
	Abuse		
☐ Yes ☐ No	Sexual	☐ Past ☐ Current	☐ Self Reported ☐ Diagnosed
☐ Yes ☐ No	Mental	☐ Past ☐ Current	☐ Self Reported ☐ Diagnosed
☐ Yes ☐ No	Physical	☐ Past ☐ Current	☐ Self Reported ☐ Diagnosed
☐ Yes ☐ No	Chronic Grief	☐ Past ☐ Current	☐ Self Reported ☐ Diagnosed
☐ Yes ☐ No	Post Traumatic Stress Disorder	☐ Past ☐ Current	☐ Self Reported ☐ Diagnosed
☐ Yes ☐ No	Binge Eating Disorder	☐ Past ☐ Current	☐ Self Reported ☐ Diagnosed
☐ Yes ☐ No	Attention Deficit Disorder	☐ Past ☐ Current	☐ Self Reported ☐ Diagnosed
☐ Yes ☐ No	Obsessive Compulsive Disorder	☐ Past ☐ Current	☐ Self Reported ☐ Diagnosed
	Addiction		
☐ Yes ☐ No	Drug	☐ Past ☐ Current	☐ Self Reported ☐ Diagnosed
☐ Yes ☐ No	Alcohol	☐ Past ☐ Current	☐ Self Reported ☐ Diagnosed
☐ Yes ☐ No	Nicotine	☐ Past ☐ Current	☐ Self Reported ☐ Diagnosed
☐ Yes ☐ No	Other		
	If Yes, specify ______________	☐ Past ☐ Current	☐ Self Reported ☐ Diagnosed
☐ Yes ☐ No	Psychosis	☐ Past ☐ Current	☐ Self Reported ☐ Diagnosed
☐ Yes ☐ No	Borderline Personality Disorder	☐ Past ☐ Current	☐ Self Reported ☐ Diagnosed

Current Medications

Is the patient **regularly** taking any **prescription** medications ☐ Yes ☐ No

If Yes, complete a ***'Prescription Medication Log'*** form

Total number of ***Prescription Medication Log*** pages faxed at this visit ________

Is the patient **regularly** taking any **over-the-counter** medications
(include vitamins & mineral supplements) ☐ Yes ☐ No

If Yes, complete an ***'OTC Medication Log'*** form

Total number of ***OTC Medication Log*** pages faxed at this visit ________

Page completed by _________ Date ____/____/_______
Initials dd mm yyyy

©2008 EPICORE Centre
Fax completed forms to the EPICORE Centre (780) 492-6059 or 1-888-215-5474

APPLES Study

Patient ID # 0 1 – ____ – ____ **Patient Initials** ___ ___ ___

Site Number Patient Number Group F M L

**Record #**<br>**Start Date** *(dd/mm/yyyy)*<br>**Name of Medication**<br>*(Generic Name Only)*	**Dose**	**Units**	**Frequency**	**Baseline**	☐ **6 Month**<br>No Change / Changed / Stopped / New	☐ **12 Month**<br>No Change / Changed / Stopped / New	☐ **18 Month**<br>No Change / Changed / Stopped / New	☐ **24 Month**<br>No Change / Changed / Stopped / New
____ __/__/____<br>__________	____	☐ mg ☐ g ☐ µg ☐ mcg ☐ IU ☐ U ☐ ml<br>☐ cc ☐ gtt ☐ Unk ☐ Other, specify ____	☐ qd ☐ bid ☐ tid ☐ qid ☐ q4h ☐ q2d<br>☐ q3d ☐ 1/wk ☐ PRN ☐ Other, specify ____	☐	☐ ☐ ☐ ☐<br>If changed or stopped:<br>Date: __/__/____<br>☐ new dose ____<br>☐ new freq ____	☐ ☐ ☐ ☐<br>If changed or stopped:<br>Date: __/__/____<br>☐ new dose ____<br>☐ new freq ____	☐ ☐ ☐ ☐<br>If changed or stopped:<br>Date: __/__/____<br>☐ new dose ____<br>☐ new freq ____	☐ ☐ ☐ ☐<br>If changed or stopped:<br>Date: __/__/____<br>☐ new dose ____<br>☐ new freq ____
____ __/__/____<br>__________	____	☐ mg ☐ g ☐ µg ☐ mcg ☐ IU ☐ U ☐ ml<br>☐ cc ☐ gtt ☐ Unk ☐ Other, specify ____	☐ qd ☐ bid ☐ tid ☐ qid ☐ q4h ☐ q2d<br>☐ q3d ☐ 1/wk ☐ PRN ☐ Other, specify ____	☐	☐ ☐ ☐ ☐<br>If changed or stopped:<br>Date: __/__/____<br>☐ new dose ____<br>☐ new freq ____	☐ ☐ ☐ ☐<br>If changed or stopped:<br>Date: __/__/____<br>☐ new dose ____<br>☐ new freq ____	☐ ☐ ☐ ☐<br>If changed or stopped:<br>Date: __/__/____<br>☐ new dose ____<br>☐ new freq ____	☐ ☐ ☐ ☐<br>If changed or stopped:<br>Date: __/__/____<br>☐ new dose ____<br>☐ new freq ____
____ __/__/____<br>__________	____	☐ mg ☐ g ☐ µg ☐ mcg ☐ IU ☐ U ☐ ml<br>☐ cc ☐ gtt ☐ Unk ☐ Other, specify ____	☐ qd ☐ bid ☐ tid ☐ qid ☐ q4h ☐ q2d<br>☐ q3d ☐ 1/wk ☐ PRN ☐ Other, specify ____	☐	☐ ☐ ☐ ☐<br>If changed or stopped:<br>Date: __/__/____<br>☐ new dose ____<br>☐ new freq ____	☐ ☐ ☐ ☐<br>If changed or stopped:<br>Date: __/__/____<br>☐ new dose ____<br>☐ new freq ____	☐ ☐ ☐ ☐<br>If changed or stopped:<br>Date: __/__/____<br>☐ new dose ____<br>☐ new freq ____	☐ ☐ ☐ ☐<br>If changed or stopped:<br>Date: __/__/____<br>☐ new dose ____<br>☐ new freq ____
____ __/__/____<br>__________	____	☐ mg ☐ g ☐ µg ☐ mcg ☐ IU ☐ U ☐ ml<br>☐ cc ☐ gtt ☐ Unk ☐ Other, specify ____	☐ qd ☐ bid ☐ tid ☐ qid ☐ q4h ☐ q2d<br>☐ q3d ☐ 1/wk ☐ PRN ☐ Other, specify ____	☐	☐ ☐ ☐ ☐<br>If changed or stopped:<br>Date: __/__/____<br>☐ new dose ____<br>☐ new freq ____	☐ ☐ ☐ ☐<br>If changed or stopped:<br>Date: __/__/____<br>☐ new dose ____<br>☐ new freq ____	☐ ☐ ☐ ☐<br>If changed or stopped:<br>Date: __/__/____<br>☐ new dose ____<br>☐ new freq ____	☐ ☐ ☐ ☐<br>If changed or stopped:<br>Date: __/__/____<br>☐ new dose ____<br>☐ new freq ____
____ __/__/____<br>__________	____	☐ mg ☐ g ☐ µg ☐ mcg ☐ IU ☐ U ☐ ml<br>☐ cc ☐ gtt ☐ Unk ☐ Other, specify ____	☐ qd ☐ bid ☐ tid ☐ qid ☐ q4h ☐ q2d<br>☐ q3d ☐ 1/wk ☐ PRN ☐ Other, specify ____	☐	☐ ☐ ☐ ☐<br>If changed or stopped:<br>Date: __/__/____<br>☐ new dose ____<br>☐ new freq ____	☐ ☐ ☐ ☐<br>If changed or stopped:<br>Date: __/__/____<br>☐ new dose ____<br>☐ new freq ____	☐ ☐ ☐ ☐<br>If changed or stopped:<br>Date: __/__/____<br>☐ new dose ____<br>☐ new freq ____	☐ ☐ ☐ ☐<br>If changed or stopped:<br>Date: __/__/____<br>☐ new dose ____<br>☐ new freq ____

Initials of person completing form ______ Date (dd/mm/yyyy) ___/___/____

©2008 EPICORE Centre

APPLES Study

Patient ID # 0 1 – ____ ____ ____ ____ – ____ ____ **Patient Initials** ____ ____ ____

Site Number / Patient Number / Group / F M L

**Record #**<br>**Start Date** *(dd/mm/yyyy)*<br>**Name of Medication**<br>*(Generic Name Only)*	**Dose**	**Units**	**Frequency**	**Baseline**	☐ **6 Month**<br>No Change / Changed / Stopped / New	☐ **12 Month**<br>No Change / Changed / Stopped / New	☐ **18 Month**<br>No Change / Changed / Stopped / New	☐ **24 Month**<br>No Change / Changed / Stopped / New
____ ___/___/_____<br>__________________	______	☐ mg ☐ g ☐ µg ☐ mcg ☐ IU ☐ U ☐ ml<br>☐ cc ☐ gtt ☐ Unk ☐ Other, specify ______	☐ qd ☐ bid ☐ tid ☐ qid ☐ q4h ☐ q2d<br>☐ q3d ☐ 1/wk ☐ PRN ☐ Other, specify ______	☐	☐ ☐ ☐ ☐<br>If changed or stopped:<br>Date: ___/___/_____<br>☐ new dose ________<br>☐ new freq ________	☐ ☐ ☐ ☐<br>If changed or stopped:<br>Date: ___/___/_____<br>☐ new dose ________<br>☐ new freq ________	☐ ☐ ☐ ☐<br>If changed or stopped:<br>Date: ___/___/_____<br>☐ new dose ________<br>☐ new freq ________	☐ ☐ ☐ ☐<br>If changed or stopped:<br>Date: ___/___/_____<br>☐ new dose ________<br>☐ new freq ________
____ ___/___/_____<br>__________________	______	☐ mg ☐ g ☐ µg ☐ mcg ☐ IU ☐ U ☐ ml<br>☐ cc ☐ gtt ☐ Unk ☐ Other, specify ______	☐ qd ☐ bid ☐ tid ☐ qid ☐ q4h ☐ q2d<br>☐ q3d ☐ 1/wk ☐ PRN ☐ Other, specify ______	☐	☐ ☐ ☐ ☐<br>If changed or stopped:<br>Date: ___/___/_____<br>☐ new dose ________<br>☐ new freq ________	☐ ☐ ☐ ☐<br>If changed or stopped:<br>Date: ___/___/_____<br>☐ new dose ________<br>☐ new freq ________	☐ ☐ ☐ ☐<br>If changed or stopped:<br>Date: ___/___/_____<br>☐ new dose ________<br>☐ new freq ________	☐ ☐ ☐ ☐<br>If changed or stopped:<br>Date: ___/___/_____<br>☐ new dose ________<br>☐ new freq ________
____ ___/___/_____<br>__________________	______	☐ mg ☐ g ☐ µg ☐ mcg ☐ IU ☐ U ☐ ml<br>☐ cc ☐ gtt ☐ Unk ☐ Other, specify ______	☐ qd ☐ bid ☐ tid ☐ qid ☐ q4h ☐ q2d<br>☐ q3d ☐ 1/wk ☐ PRN ☐ Other, specify ______	☐	☐ ☐ ☐ ☐<br>If changed or stopped:<br>Date: ___/___/_____<br>☐ new dose ________<br>☐ new freq ________	☐ ☐ ☐ ☐<br>If changed or stopped:<br>Date: ___/___/_____<br>☐ new dose ________<br>☐ new freq ________	☐ ☐ ☐ ☐<br>If changed or stopped:<br>Date: ___/___/_____<br>☐ new dose ________<br>☐ new freq ________	☐ ☐ ☐ ☐<br>If changed or stopped:<br>Date: ___/___/_____<br>☐ new dose ________<br>☐ new freq ________
____ ___/___/_____<br>__________________	______	☐ mg ☐ g ☐ µg ☐ mcg ☐ IU ☐ U ☐ ml<br>☐ cc ☐ gtt ☐ Unk ☐ Other, specify ______	☐ qd ☐ bid ☐ tid ☐ qid ☐ q4h ☐ q2d<br>☐ q3d ☐ 1/wk ☐ PRN ☐ Other, specify ______	☐	☐ ☐ ☐ ☐<br>If changed or stopped:<br>Date: ___/___/_____<br>☐ new dose ________<br>☐ new freq ________	☐ ☐ ☐ ☐<br>If changed or stopped:<br>Date: ___/___/_____<br>☐ new dose ________<br>☐ new freq ________	☐ ☐ ☐ ☐<br>If changed or stopped:<br>Date: ___/___/_____<br>☐ new dose ________<br>☐ new freq ________	☐ ☐ ☐ ☐<br>If changed or stopped:<br>Date: ___/___/_____<br>☐ new dose ________<br>☐ new freq ________
____ ___/___/_____<br>__________________	______	☐ mg ☐ g ☐ µg ☐ mcg ☐ IU ☐ U ☐ ml<br>☐ cc ☐ gtt ☐ Unk ☐ Other, specify ______	☐ qd ☐ bid ☐ tid ☐ qid ☐ q4h ☐ q2d<br>☐ q3d ☐ 1/wk ☐ PRN ☐ Other, specify ______	☐	☐ ☐ ☐ ☐<br>If changed or stopped:<br>Date: ___/___/_____<br>☐ new dose ________<br>☐ new freq ________	☐ ☐ ☐ ☐<br>If changed or stopped:<br>Date: ___/___/_____<br>☐ new dose ________<br>☐ new freq ________	☐ ☐ ☐ ☐<br>If changed or stopped:<br>Date: ___/___/_____<br>☐ new dose ________<br>☐ new freq ________	☐ ☐ ☐ ☐<br>If changed or stopped:<br>Date: ___/___/_____<br>☐ new dose ________<br>☐ new freq ________

Initials of person completing form __________ Date (dd/mm/yyyy) ____/____/________

Fax completed forms to the EPICORE Centre (780) 492-6059 or 1-888-215-5474

©2008 EPICORE Centre

APPLES Study

Patient ID # 0 1 – ____ – ____ **Patient Initials** ___ ___ ___

Site Number · Patient Number · Group · F M L

Record Number	Name of OTC Medication	Baseline	6 Month: Continuing	6 Month: Stopped	6 Month: New	6 Month: Estimated Cost (Round to nearest $)	12 Month: Continuing	12 Month: Stopped	12 Month: New	12 Month: Estimated Cost (Round to nearest $)	18 Month: Continuing	18 Month: Stopped	18 Month: New	18 Month: Estimated Cost (Round to nearest $)	24 Month: Continuing	24 Month: Stopped	24 Month: New	24 Month: Estimated Cost (Round to nearest $)
#____		☐	☐	☐	☐	$____.00	☐	☐	☐	$____.00	☐	☐	☐	$____.00	☐	☐	☐	$____.00
#____		☐	☐	☐	☐	$____.00	☐	☐	☐	$____.00	☐	☐	☐	$____.00	☐	☐	☐	$____.00
#____		☐	☐	☐	☐	$____.00	☐	☐	☐	$____.00	☐	☐	☐	$____.00	☐	☐	☐	$____.00
#____		☐	☐	☐	☐	$____.00	☐	☐	☐	$____.00	☐	☐	☐	$____.00	☐	☐	☐	$____.00
#____		☐	☐	☐	☐	$____.00	☐	☐	☐	$____.00	☐	☐	☐	$____.00	☐	☐	☐	$____.00
#____		☐	☐	☐	☐	$____.00	☐	☐	☐	$____.00	☐	☐	☐	$____.00	☐	☐	☐	$____.00
#____		☐	☐	☐	☐	$____.00	☐	☐	☐	$____.00	☐	☐	☐	$____.00	☐	☐	☐	$____.00
#____		☐	☐	☐	☐	$____.00	☐	☐	☐	$____.00	☐	☐	☐	$____.00	☐	☐	☐	$____.00
#____		☐	☐	☐	☐	$____.00	☐	☐	☐	$____.00	☐	☐	☐	$____.00	☐	☐	☐	$____.00
#____		☐	☐	☐	☐	$____.00	☐	☐	☐	$____.00	☐	☐	☐	$____.00	☐	☐	☐	$____.00
#____		☐	☐	☐	☐	$____.00	☐	☐	☐	$____.00	☐	☐	☐	$____.00	☐	☐	☐	$____.00
#____		☐	☐	☐	☐	$____.00	☐	☐	☐	$____.00	☐	☐	☐	$____.00	☐	☐	☐	$____.00

Initials of person completing form ________ Date (dd/mm/yyyy) ____/____/______

©2008 EPICORE Centre

Fax completed forms to EPICORE Centre (780) 492-6059 or 1-888-215-5474

APPLES Study

Patient ID # 0 1 – ____ – ____ **Patient Initials** ___ ___ ___

Site Number | Patient Number | Group | F M L

Record Number	Name of OTC Medication	Baseline	☐ 6 Month				☐ 12 Month				☐ 18 Month				☐ 24 Month			
			Continuing	Stopped	New	Estimated Cost (Round to nearest $)	Continuing	Stopped	New	Estimated Cost (Round to nearest $)	Continuing	Stopped	New	Estimated Cost (Round to nearest $)	Continuing	Stopped	New	Estimated Cost (Round to nearest $)
#____		☐	☐	☐	☐	$ ____.00	☐	☐	☐	$ ____.00	☐	☐	☐	$ ____.00	☐	☐	☐	$ ____.00
#____		☐	☐	☐	☐	$ ____.00	☐	☐	☐	$ ____.00	☐	☐	☐	$ ____.00	☐	☐	☐	$ ____.00
#____		☐	☐	☐	☐	$ ____.00	☐	☐	☐	$ ____.00	☐	☐	☐	$ ____.00	☐	☐	☐	$ ____.00
#____		☐	☐	☐	☐	$ ____.00	☐	☐	☐	$ ____.00	☐	☐	☐	$ ____.00	☐	☐	☐	$ ____.00
#____		☐	☐	☐	☐	$ ____.00	☐	☐	☐	$ ____.00	☐	☐	☐	$ ____.00	☐	☐	☐	$ ____.00
#____		☐	☐	☐	☐	$ ____.00	☐	☐	☐	$ ____.00	☐	☐	☐	$ ____.00	☐	☐	☐	$ ____.00
#____		☐	☐	☐	☐	$ ____.00	☐	☐	☐	$ ____.00	☐	☐	☐	$ ____.00	☐	☐	☐	$ ____.00
#____		☐	☐	☐	☐	$ ____.00	☐	☐	☐	$ ____.00	☐	☐	☐	$ ____.00	☐	☐	☐	$ ____.00
#____		☐	☐	☐	☐	$ ____.00	☐	☐	☐	$ ____.00	☐	☐	☐	$ ____.00	☐	☐	☐	$ ____.00
#____		☐	☐	☐	☐	$ ____.00	☐	☐	☐	$ ____.00	☐	☐	☐	$ ____.00	☐	☐	☐	$ ____.00
#____		☐	☐	☐	☐	$ ____.00	☐	☐	☐	$ ____.00	☐	☐	☐	$ ____.00	☐	☐	☐	$ ____.00
#____		☐	☐	☐	☐	$ ____.00	☐	☐	☐	$ ____.00	☐	☐	☐	$ ____.00	☐	☐	☐	$ ____.00

Initials of person completing form ________ Date (dd/mm/yyyy) ____/____/______

©2008 EPICORE Centre

Fax completed forms to EPICORE Centre (780) 492-6059 or 1-888-215-5474

APPLES Study

Patient ID #	0	1	–					–			Patient Initials			
	Site Number			Patient Number					Group			F	M	L

Laboratory

Date of Collection ___ /___ /________
dd mm yyyy

Lab Not Available	Lab Test	Lab Value	Unit of Collection	Same Date of Collection	If Date of Collection is different from above, enter below *(dd/mm/yyyy)*
☐	HbA1c	___ • ___	%	☐	____/____/________
☐	Glucose *(Fasting)*	___ ___ • ___	mmol/L	☐	____/____/________
☐	Insulin	___ ___ • ___	mU/L	☐	____/____/________
☐	Creatinine	___ ___ ___	µmol/L	☐	____/____/________
☐	GFR *(Calculated)*	___ ___	mL/min/1.73/m^2	☐	____/____/________
	Fasting Lipid Panel				
☐	Total Cholesterol	__ __ • __ __	mmol/L	☐	____/____/________
☐	LDL	__ __ • __ __	mmol/L	☐	____/____/________
☐	HDL	__ __ • __ __	mmol/L	☐	____/____/________
☐	Triglycerides	__ __ • __ __	mmol/L	☐	____/____/________
☐	CRP	___ ___ • ___	mg/L	☐	____/____/________
☐	GGT	___ ___ ___	U/L	☐	____/____/________
☐	Albumin	___ ___	g/L	☐	____/____/________
☐	TBIL	___ ___ ___	µmol/L	☐	____/____/________
☐	ALP	___ ___ ___	U/L	☐	____/____/________
☐	ALT	___ ___ ___	U/L	☐	____/____/________
☐	Total Protein	___ ___ ___	g/L	☐	____/____/________
☐	Ferritin	___ ___ ___	µg/L	☐	____/____/________
☐	UALB/CR	__ __ • __ __	mg/mmol	☐	____/____/________
☐	Hemoglobin	___ ___ ___	g/L	☐	____/____/________
☐	MCV	___ ___ ___	fL	☐	____/____/________
☐	Uric Acid *(Urate)*	___ ___ ___	µmol/L	☐	____/____/________
☐	TSH	___ • ___ ___	mU/L	☐	____/____/________
☐	PTH	___ ___ • ___	pmol/L	☐	____/____/________
☐	Vitamin D3	__ __ __ __ __	nmol/L	☐	____/____/________
☐	Vitamin B12	__ __ __	pmol/L	☐	____/____/________

Page completed by ________ Date ____/____/______
Initials dd mm yyyy

©2008 EPICORE Centre

Fax completed forms to the EPICORE Centre (780) 492-6059 or 1-888-215-5474

APPLES Study

Patient ID # 0 1 – ____ – ____ **Patient Initials** ___ ___ ___

Site Number — Patient Number — Group — F M L

Physical Assessment

Blood Pressure (Sitting) ___ ___ ___ / ___ ___ ___ mmHg (systolic / diastolic) Heart Rate ________ bpm

Is the patient pregnant ☐ Yes ☐ No ☐ N/A *(Male)*

Anthropometric Measures

Weight ________ kg Height ________ cm

Not for Data Entry - For Research Group Use Only ________ / ________ = ________

Weight in kg / (Height in m)^2 = BMI (kg/m^2)

Activity and Food

Average steps/day *(Pedometer)* ________ Keeping food record ☐ Yes ☐ No

Smoking History

☐ Current Smoker *(i.e. smoking (now or in the past 12 months)* ☐ Former Smoker ☐ Never Smoked

Edmonton Obesity Staging System

Score ☐ 0 ☐ 1 ☐ 2 ☐ 3 ☐ 4

Employment Status

In the past year, did you work for pay at a job or in a business *(Include all part-time jobs, seasonal work, contract work, self employment, baby-sitting and any other* ***paid*** *work regardless of the number of hours worked).* ☐ Yes ☐ No ☐ N/D

If Yes, **how many weeks** did you do any work at your jobs/businesses ______ *(weeks)* ☐ N/D
(Include paid vacation leave, paid maternity leave and paid sick leave)

About **how many hours a week** did you usually work at your jobs or business? ______ *(hours)* ☐ N/D
(If you usually work extra hours, paid or unpaid, please include those hours)

In the past 6 months, **how many days, or part days,** of work have you missed due to illness or medical problems ______ *(days)* ☐ N/D

Page completed by ________ (Initials) Date ____/____/______ (dd mm yyyy)

©2008 EPICORE Centre
Fax completed forms to the EPICORE Centre (780) 492-6059 or 1-888-215-5474

APPLES Study

Patient ID # 0 1 – ____ – ____ **Patient Initials** ___ ___ ___

Site Number | Patient Number | Group | F M L

Economic Impact

In the past year, did you receive any of the following benefits

☐ Yes ☐ No Employment Insurance or Sick Leave

If Yes, for how many weeks ________

What amount per week $ ____________ . 00 ☐ Unknown

☐ Yes ☐ No Disability Benefits

If Yes, for how many weeks ________

What amount per week $ ____________ . 00 ☐ Unknown

☐ Yes ☐ No Assured Income for the Severely Handicapped *(AISH)*

If Yes, for how many weeks ________

What amount per week $ ____________ . 00 ☐ Unknown

In the past year, did you **purchase** any weight loss intervention products or programs ☐ Yes ☐ No

If Yes, check all that apply	Estimated total cost including insurance or other payer ($ only)	Estimated out-of-pocket costs ($ only)
☐ Meal Replacements	$ __________.00	$ __________.00
☐ Physical Trainer	$ __________.00	$ __________.00
☐ Exercise Program	$ __________.00	$ __________.00
☐ **Alternative** Medicine/Therapies *(eg. acupuncture/herbs)*	$ __________.00	$ __________.00
☐ OTC **weight loss** medications	$ __________.00	$ __________.00
☐ Nutrition Counselling	$ __________.00	$ __________.00
☐ Commercial Program/Popular Diet *(eg. Weight Watchers)*	$ __________.00	$ __________.00
☐ Other, specify ______________________	$ __________.00	$ __________.00

Page completed by _________ Date ____/____/______
Initials dd mm yyyy

©2008 EPICORE Centre
Fax completed forms to the EPICORE Centre (780) 492-6059 or 1-888-215-5474

APPLES Study

Patient ID #	0 1	–		–		Patient Initials			
	Site Number		Patient Number		Group		F	M	L

In the past 6 months, did you receive or acquire any mobility/medical aids/services ☐ Yes ☐ No

If Yes, check all that apply	Estimated total cost including insurance or other payer *($ only)*	Estimated out-of-pocket costs *($ only)*
☐ Mobility Aids *(walker/cane/scooter)*	$ ________.00	$ ________.00
☐ Home modification/renovations *(ramps/bars/rails)*	$ ________.00	$ ________.00
☐ Personal care assistance *(washing/bathing/wound care)*	$ ________.00	$ ________.00
☐ Household care assistance *(laundry/cooking/cleaning)*	$ ________.00	$ ________.00
☐ Transport assistance *(driver/taxi/DATS)*	$ ________.00	$ ________.00
☐ Other, specify ____________________	$ ________.00	$ ________.00

Home Care/LTC

In the past year, did you receive any assistance from the following professionals *(eg. help with personal care, washing, etc.)* ☐ Yes ☐ No

If Yes, check all that apply	Provided through paid service	Total # of hours in reporting period	Estimated total cost including insurance or other payer *($ only)*	Estimated out-of-pocket costs *($ only)*
☐ Nurse	☐ Yes ☐ No	______	$ ________.00	$ ________.00
☐ Physiotherapist	☐ Yes ☐ No	______	$ ________.00	$ ________.00
☐ Occupational Therapist	☐ Yes ☐ No	______	$ ________.00	$ ________.00
☐ Respiratory Therapist	☐ Yes ☐ No	______	$ ________.00	$ ________.00
☐ Other	☐ Yes ☐ No	______	$ ________.00	$ ________.00

If Yes, specify ____________________

Page completed by ________ Date ____/____/______
Initials dd mm yyyy

©2008 EPICORE Centre

Fax completed forms to the EPICORE Centre (780) 492-6059 or 1-888-215-5474

APPLES Study

Patient ID # 0 1 – ____ – ____ **Patient Initials** ___ ___ ___

Site Number — Patient Number — Group — F M L

Questionnaires

Have the following questionnaires been administered

☐ Yes	☐ No	**SF-12**	If Yes, Date ____ / ____ / ________ (dd / mm / yyyy)	______ Initials
☐ Yes	☐ No	**EQ-5D**	If Yes, Date ____ / ____ / ________ (dd / mm / yyyy)	______ Initials
☐ Yes	☐ No	**IWQoL-Lite**	If Yes, Date ____ / ____ / ________ (dd / mm / yyyy)	______ Initials
☐ Yes	☐ No	**PSS**	If Yes, Date ____ / ____ / ________ (dd / mm / yyyy)	______ Initials

The following questionnaire is to be administered ONLY to patients in the *Community Wait List* group

☐ Yes	☐ No	**Modified WLIQ**	If Yes, Date ____ / ____ / ________ (dd / mm / yyyy)	______ Initials

Page completed by ________ Initials Date ____/____/______ dd mm yyyy

©2008 EPICORE Centre
Fax completed forms to the EPICORE Centre (780) 492-6059 or 1-888-215-5474

APPLES Study

Patient ID # | 0 | 1 | – | | | | | – | | | **Patient Initials** | | | |

Site Number — Patient Number — Group — F M L

Resource Utilization

In the past year, were any of the following resources utilized ☐ Yes ☐ No

If Yes, *check all that apply*

- ☐ ED visit *(without hospital admission)* How many ________
- ☐ Hospital admission *(including overnight stay)* How many ________
- ☐ GP visit How many ________

Page completed by ________ Date ____/____/______
Initials dd mm yyyy

©2009 EPICORE Centre

APPLES Study

Questionnaire Package

Dear Study Participant

These questionnaires ask about your health. Please select the answer that best describes how you feel. There are no right or wrong answers. No matter what answers you record, you are guaranteed the same treatment. You may leave an answer blank if you object to the question. If you have any questions or concerns, you may direct them to the research staff.

The information you provide will be kept confidential. Only the investigators will have access to the information collected in this study. Any report or presentation of this study will not identify you by name.

Thank you for your time

©2008 EPICORE Centre

APPLES Study

Patient ID #	0	1	–					–			Patient Initials			
	Site Number			Patient Number					Group			F	M	L

Visit ☐ **Baseline** ☐ **6 Month** ☐ **12 Month** ☐ **18 Month** ☐ **24 Month**

This survey asks for your views about your health. This information will help keep track of how you feel and how well you are able to do your usual activities. For each of the following questions, please mark the one checkbox that best describes your answer.

1. In general, would you say your health is:

☐ Excellent ☐ Very good ☐ Good ☐ Fair ☐ Poor

2. The following questions are about activities you might do during a typical day. Does your health now limit you in these activities? If so, how much?

a. Moderate activities, such as moving a table, pushing a vacuum cleaner, bowling or playing golf

☐ Yes, limited a lot ☐ Yes, limited a little ☐ No, not limited at all

b. Climbing several flights of stairs

☐ Yes, limited a lot ☐ Yes, limited a little ☐ No, not limited at all

3. During the past 4 weeks, how much of the time have you had any of the following problems with your work or other regular daily activities as a result of your physical health?

a. Accomplished less than you would like

☐ All of the time ☐ Most of the time ☐ Some of the time ☐ A little of the time ☐ None of the time

b. Were limited in the kind of work or other activities

☐ All of the time ☐ Most of the time ☐ Some of the time ☐ A little of the time ☐ None of the time

4. During the past 4 weeks, how much of the time have you had any of the following problems with your work or other regular daily activities as a result of any emotional problems (such as feeling depressed or anxious)?

a. Accomplished less than you would like

☐ All of the time ☐ Most of the time ☐ Some of the time ☐ A little of the time ☐ None of the time

b. Did work or other activities less carefully than usual

☐ All of the time ☐ Most of the time ☐ Some of the time ☐ A little of the time ☐ None of the time

SF-12v2™ Health Survey © 1994, 2002 QualityMetric Incorporated and Medical Outcomes Trust. All rights reserved

©2008 EPICORE Centre

Fax completed forms to the EPICORE Centre (780) 492-6059 or 1-888-215-5474

APPLES Study

Patient ID # 0 1 – ☐☐☐☐ – ☐☐ **Patient Initials** ☐☐☐

Site Number — Patient Number — Group — F M L

Visit ☐ **Baseline** ☐ **6 Month** ☐ **12 Month** ☐ **18 Month** ☐ **24 Month**

5. During the past 4 weeks, how much did pain interfere with your normal work (including both work outside the home and housework)?

☐ Not at all ☐ A little bit ☐ Moderately ☐ Quite a bit ☐ Extremely

6. These questions are about how you feel and how things have been with you during the past 4 weeks. For each question, please give the one answer that comes closest to the way you have been feeling. How much of the time during the past 4 weeks ...

a. Have you felt calm and peaceful?

☐ All of the time ☐ Most of the time ☐ Some of the time ☐ A little of the time ☐ None of the time

b. Did you have a lot of energy?

☐ All of the time ☐ Most of the time ☐ Some of the time ☐ A little of the time ☐ None of the time

c. Have felt downhearted and depressed?

☐ All of the time ☐ Most of the time ☐ Some of the time ☐ A little of the time ☐ None of the time

7. During the past 4 weeks, how much of the time has your physical health or emotional problems interfered with your social activities (like visiting with friends, relatives, etc.)?

☐ All of the time ☐ Most of the time ☐ Some of the time ☐ A little of the time ☐ None of the time

Date questionnaire completed ______/_______/________
day month year

SF-12v2™ Health Survey © 1994, 2002 QualityMetric Incorporated and Medical Outcomes Trust. All rights reserved

©2008 EPICORE Centre

APPLES Study

Patient ID #	0	1	–					–			Patient Initials			
	Site Number			Patient Number					Group			F	M	L

Visit	☐	Baseline	☐	6 Month	☐	12 Month	☐	18 Month	☐	24 Month

By marking one box in each group below, please indicate which statements best describe your own state of health today.

Mobility

I have no problems in walking about ☐
I have some problems in walking about ☐
I am confined to bed ☐

Self-Care

I have no problems with self care ☐
I have some problems washing or dressing myself ☐
I am unable to wash or dress myself ☐

Usual Activities *(eg. work, study, housework, family or leisure activities)*

I have no problems with performing my usual activities ☐
I have some problems with performing my usual activities ☐
I am unable to perform my usual activities ☐

Pain/Discomfort

I have no pain or discomfort ☐
I have moderate pain or discomfort ☐
I have extreme pain or discomfort ☐

Anxiety/Depression

I am not anxious or depressed ☐
I am moderately anxious or depressed ☐
I am extremely anxious or depressed ☐

©2008 EPICORE Centre
Fax completed forms to the EPICORE Centre (780) 492-6059 or 1-888-215-5474

APPLES Study

**Patient ID #**	0	1	–					–			**Patient Initials**			
	Site Number			Patient Number					Group			F	M	L

**Visit**		**Baseline**		**6 Month**		**12 Month**		**18 Month**		**24 Month**

To help people say how good or bad their state of health is, we use this scale on which the best state you can imagine is marked 100 and the worst state you can imagine is marked 0. I would like you to indicate on this scale how good or bad your own health is today, in your opinion. Please do this by drawing a line from this box to whichever point on the scale indicates how good or bad your state of health is today."

Your own state of health today

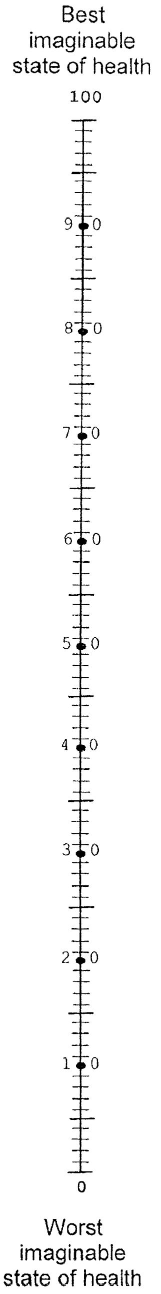

Date questionnaire completed ______ / ______ / ___________
day month year

For Study Coordinator Use Only

___ ___ ___
State of Health Score

©2008 EPICORE Centre

Fax completed forms to the EPICORE Centre (780) 492-6059 or 1-888-215-5474

APPLES Study

Patient ID #	0	1	–					–			Patient Initials			
	Site Number			Patient Number					Group			F	M	L

Visit ☐ **Baseline** ☐ **6 Month** ☐ **12 Month** ☐ **18 Month** ☐ **24 Month**

Please answer the following statements by marking the box under the statement that best applies to you in the past week. Be as open as possible. There are no right or wrong answers.

(Check one box ONLY on each line)

		Always True	Usually True	Sometimes True	Rarely True	Never True
**Physical Function**						
1.	Because of my weight I have trouble picking up objects.	☐	☐	☐	☐	☐
2.	Because of my weight I have trouble tying my shoes.	☐	☐	☐	☐	☐
3.	Because of my weight I have difficulty getting up from chairs.	☐	☐	☐	☐	☐
4.	Because of my weight I have trouble using stairs.	☐	☐	☐	☐	☐
5.	Because of my weight I have difficulty putting on or taking off my clothing.	☐	☐	☐	☐	☐
6.	Because of my weight I have trouble with mobility.	☐	☐	☐	☐	☐
7.	Because of my weight I have trouble crossing my legs.	☐	☐	☐	☐	☐
8.	I feel short of breath with only mild exertion.	☐	☐	☐	☐	☐
9.	I am troubled by painful or stiff joints.	☐	☐	☐	☐	☐
10.	My ankles and lower legs are swollen at the end of the day.	☐	☐	☐	☐	☐
11.	I am worried about my health.	☐	☐	☐	☐	☐
**Self-esteem**						
1.	Because of my weight I am self-conscious.	☐	☐	☐	☐	☐
2.	Because of my weight my self-esteem is not what it could be.	☐	☐	☐	☐	☐
3.	Because of my weight I feel unsure of myself.	☐	☐	☐	☐	☐
4.	Because of my weight I don't like myself.	☐	☐	☐	☐	☐
5.	Because of my weight I am afraid of being rejected.	☐	☐	☐	☐	☐
6.	Because of my weight I avoid looking in mirrors or seeing myself in photographs.	☐	☐	☐	☐	☐
7.	Because of my weight I am embarrassed to be seen in public places.	☐	☐	☐	☐	☐

©Copyright 2000. Duke University Medical Center

©2008 EPICORE Centre

Fax completed forms to the EPICORE Centre (780) 492-6059 or 1-888-215-5474

APPLES Study

Patient ID #	0	1	–					–			Patient Initials			
	Site Number			Patient Number					Group			F	M	L

Visit	☐ Baseline	☐ 6 Month	☐ 12 Month	☐ 18 Month	☐ 24 Month

(Check one box ONLY on each line)

		Always True	Usually True	Sometimes True	Rarely True	Never True
**Sexual Life**						
1.	Because of my weight I do not enjoy sexual activity.	☐	☐	☐	☐	☐
2.	Because of my weight I have little or no sexual desire.	☐	☐	☐	☐	☐
3.	Because of my weight I have difficulty with sexual performance.	☐	☐	☐	☐	☐
4.	Because of my weight I avoid sexual encounters whenever possible.	☐	☐	☐	☐	☐
**Public Distress**						
1.	Because of my weight I experience ridicule, teasing, or unwanted attention.	☐	☐	☐	☐	☐
2.	Because of my weight I worry about fitting into seats in public places (e.g. theatres, restaurants, cars, or airplanes).	☐	☐	☐	☐	☐
3.	Because of my weight I worry about fitting through aisles or turnstiles.	☐	☐	☐	☐	☐
4.	Because of my weight I worry about finding chairs that are strong enough to hold my weight.	☐	☐	☐	☐	☐
5.	Because of my weight I experience discrimination by others.	☐	☐	☐	☐	☐
**Work** (Note: For homemakers and retirees, answer with respect to your daily activities)						
1.	Because of my weight I have trouble getting things accomplished or meeting my responsibilities.	☐	☐	☐	☐	☐
2.	Because of my weight I am less productive than I could be.	☐	☐	☐	☐	☐
3.	Because of my weight I don't receive appropriate raises, promotions or recognition at work.	☐	☐	☐	☐	☐
4.	Because of my weight I am afraid to go on job interviews.	☐	☐	☐	☐	☐

Date questionnaire completed _______/_________/_________
day month year

©Copyright 2000. Duke University Medical Center

©2008 EPICORE Centre

Fax completed forms to the EPICORE Centre (780) 492-6059 or 1-888-215-5474

APPLES Study

**Patient ID #**	0 1	–		–		**Patient Initials**			
	Site Number		Patient Number		Group		F	M	L

**Visit**	☐ **Baseline**	☐ **6 Month**	☐ **12 Month**	☐ **18 Month**	☐ **24 Month**

We would like to find out how satisfied you have felt with the medical care you have been receiving since you were referred to the Weight Wise program.

How strongly do you AGREE or DISAGREE with each of the following statements? On the line beside each statement, mark the checkbox below the opinion which is closest to your own view.

(Check one box ONLY on each line)

		**Strongly Agree**	**Agree**	**Uncertain**	**Disagree**	**Strongly Disagree**
1.	The medical care I have been receiving is just about perfect	☐	☐	☐	☐	☐
2.	I am dissatisfied with some things about the medical care I received	☐	☐	☐	☐	☐

Date questionnaire completed _______ /_________ /_________
day month year

©2008 EPICORE Centre
Fax completed forms to the EPICORE Centre (780) 492-6059 or 1-888-215-5474

APPLES Study

Patient ID #	0 1	–		–		Patient Initials			
	Site Number		Patient Number		Group		F	M	L

Visit	☐ Baseline	☐ 6 Month	☐ 12 Month	☐ 18 Month	☐ 24 Month

How strongly do you AGREE or DISAGREE with <u>each</u> of the following statements? On the line beside each statement, mark the checkbox below the opinion which is closest to your own view.

(Check one box ONLY on each line)

	Strongly Agree	Agree	Neutral or not relevant	Disagree	Strongly Disagree
**Employment Issues**					
Because of my weight problem, I am unable to work	☐	☐	☐	☐	☐
Because of my weight problem, I can't work a full shift	☐	☐	☐	☐	☐
Money is a great issue for me now	☐	☐	☐	☐	☐
**Physical Stress**					
I make sure I don't overdo things	☐	☐	☐	☐	☐
Physical activities take me longer now	☐	☐	☐	☐	☐
My activity is reduced because of my obesity	☐	☐	☐	☐	☐
I can't do many of the things I used to do	☐	☐	☐	☐	☐
I am very short of breath	☐	☐	☐	☐	☐
I have angina	☐	☐	☐	☐	☐
The rest of my body is suffering because of my weight condition	☐	☐	☐	☐	☐
My symptoms are getting worse	☐	☐	☐	☐	☐
I am feeling fine now	☐	☐	☐	☐	☐
I'm not sure what activity I can do without hurting my condition	☐	☐	☐	☐	☐
I've stopped smoking recently	☐	☐	☐	☐	☐
**Social Support**					
My family and friends are very patient and supportive	☐	☐	☐	☐	☐
I try to cope	☐	☐	☐	☐	☐
I have faith in the doctors	☐	☐	☐	☐	☐
Waiting is very tough on my family and friends	☐	☐	☐	☐	☐
I would attend a support group for people with obesity	☐	☐	☐	☐	☐
A big factor is the lack of communication in the system	☐	☐	☐	☐	☐
I would attend a class to learn more about obesity	☐	☐	☐	☐	☐

©2008 EPICORE Centre

Fax completed forms to the EPICORE Centre (780) 492-6059 or 1-888-215-5474

APPLES Study

Patient ID # 0 1 – ☐☐☐☐ – ☐☐ Patient Initials ☐☐☐

Site Number / Patient Number / Group / F M L

Visit ☐ Baseline ☐ 6 Month ☐ 12 Month ☐ 18 Month ☐ 24 Month

(Check one box ONLY on each line)

	Strongly Agree	Agree	Neutral or not relevant	Disagree	Strongly Disagree
**Frustration**					
It frustrates me that I have to wait for obesity treatment	☐	☐	☐	☐	☐
I worry about what might happen while waiting *(e.g. worsening symptoms, heart attack, death)*	☐	☐	☐	☐	☐
I'm frustrated with the allocation of resources	☐	☐	☐	☐	☐
The problem with the waiting list is the allocation of resources	☐	☐	☐	☐	☐
I am mad and upset about the wait	☐	☐	☐	☐	☐
I am scared of treatment for obesity	☐	☐	☐	☐	☐
I am afraid to go away from the phone for too long in case I miss a call for obesity treatment	☐	☐	☐	☐	☐
I am interested in obesity surgery	☐	☐	☐	☐	☐
The waiting list is not fair to everybody	☐	☐	☐	☐	☐
I shouldn't have to wait for obesity treatment	☐	☐	☐	☐	☐
**Quality of Life**					
I have no control over the situation	☐	☐	☐	☐	☐
I just want to get it over with	☐	☐	☐	☐	☐
Waiting has affected my quality of life	☐	☐	☐	☐	☐
The length of waiting is a big concern	☐	☐	☐	☐	☐
It is very stressful waiting for obesity treatment	☐	☐	☐	☐	☐
My life has been put on hold while I wait for obesity treatment	☐	☐	☐	☐	☐
Waiting costs you physically, mentally and financially	☐	☐	☐	☐	☐
I am anxious and worried about treatment	☐	☐	☐	☐	☐
There is no quality of life while waiting for treatment	☐	☐	☐	☐	☐

Date questionnaire completed _______/_________/_________
day month year

©2008 EPICORE Centre
Fax completed forms to the EPICORE Centre (780) 492-6059 or 1-888-215-5474
